# Omicron XBB.1.5 subvariant causes severe pulmonary disease in K18-hACE-2 mice

**DOI:** 10.3389/fmicb.2024.1466980

**Published:** 2024-10-02

**Authors:** Amany Elsharkawy, Shannon Stone, Anchala Guglani, Lila D. Patterson, Chunyu Ge, Chinonye Dim, Joseph M. Miano, Mukesh Kumar

**Affiliations:** ^1^Department of Biology, College of Arts and Sciences, Georgia State University, Atlanta, GA, United States; ^2^Center of Diagnostics and Therapeutics, Georgia State University, Atlanta, GA, United States; ^3^Vascular Biology Center, Medical College of Georgia at Augusta University, Augusta, GA, United States

**Keywords:** COVID-19, SARS-CoV-2 variants of concern, XBB.1.5, inflammation, ACE-2 expressing mice

## Abstract

Owing to their continuous evolution, severe acute respiratory syndrome coronavirus 2 (SARS-CoV-2) variants of concern (VOCs) display disparate pathogenicity in mouse models. Omicron and its sublineages have been dominant worldwide. Compared to pre-Omicron VOCs, early Omicron subvariants reportedly cause attenuated disease in human ACE-2-expressing mice (K18-hACE-2). In late 2022, the frequency of Omicron subvariant XBB.1.5 rapidly increased and it progressively replaced other circulating strains. The emergence of new strains requires current SARS-CoV-2 clinical animal model re-evaluation. In this study, we aim to characterize XBB.1.5 pathogenesis in K18-hACE-2. Herein, we demonstrated that XBB.1.5 infection is associated with significant weight loss, severe lung pathology, and substantial mortality. Intranasal XBB.1.5 infection resulted in 100% mortality in K18-hACE2 mice. High virus titers were detected in the lungs on days 3 and 5 after infection. Moreover, XBB.1.5 productively infected the cells within the nasal turbinate, olfactory bulb, intestines, and kidneys. In addition, in a subset of infected mice, we detected high virus titers in the brain. Consistently, we detected high viral antigen expression in the lungs. Furthermore, we observed severe lung injury hallmarks (e.g., immune cell infiltration, perivascular cuffing, and alveolar consolidation). Using immunofluorescence labeling and cytometric analysis, we revealed that XBB.1.5 infection leads to CD45^+^ cell influx into the lung parenchyma. We further demonstrated that most immune infiltrates are CD11b^+^ CD11c^+^ dendritic cells. Additionally, we detected significant induction of proinflammatory cytokines and chemokines in infected lungs. Taken together, our data show that Omicron subvariant XBB.1.5 is highly pathogenic in K18-hACE2 mice.

## 1 Introduction

Since the emergence of severe acute respiratory syndrome coronavirus 2 (SARS-CoV-2) in late 2019, variants of concerns (VOCs) have spurred recurring global infection waves. VOCs confer notable mutations in the S gene with the potential for enhanced transmission, disease severity, virulence, and immune escape (Zhou P. et al., 2020; Jhun et al., 2021; Martínez-Flores et al., 2021). The Omicron variant was first reported in November 2021 in South Africa and is the most mutated SARS-CoV-2 variant with a significant number of mutations. Omicron displays more than 30 amino acid substitutions, deletions, and an insertion in the S protein compared to the ancestral strain, contributing to its increased transmissibility and immune evasion properties. Such properties are attributed to reduced spike cleavage and attenuated pathogenicity (Fan et al., 2022; Kumar et al., 2022). Omicron gradually evolved into several sublineages, i.e., BA.1–5, becoming globally dominant (Kurhade et al., 2023; Uraki et al., 2023). Several studies reported the reduced pathogenicity of the BA.1 and BA.2 sublineages compared to pre-Omicron VOCs (Nyberg et al., 2022; Ward et al., 2022). Recently, two Omicron subvariants, BQ.1 and XBB, progressively replaced other Omicron subvariants (Wang et al., 2023). XBB is a recombinant of variants BA.2.10.1 and BA.2.75, two offshoots of the Omicron BA.2 lineage. Recently, the XBB lineages, including XBB.1 and XBB.1.5, have become a global public health concern due to their enhanced transmissibility, infectivity, and immune resistance. XBB strains are extremely evasive against therapeutic monoclonal antibodies and immunity induced by prior vaccines or natural infections (Uriu et al., 2023; Yue et al., 2023). XBB.1.5 was first identified in New York, in the United States, in October 2022 and has emerged as the dominant strain in several countries. Similar to XBB.1, XBB.1.5 harbors the G252V substitution mutation. In addition, XBB1.5 carries a unique F486P substitution mutation, contributing to its significantly higher RBD-human angiotensin-converting enzyme 2 (hACE2) binding affinity and superior transmissibility compared to other strains (Yue et al., 2023).

The emergence of Omicron XBB subvariants has significantly reduced COVID-19 vaccine efficacy including that of the original monovalent and the WA1/BA.5 bivalent mRNA vaccines (Wang et al., 2023). Consequently, COVID-19 vaccines have been updated to better target XBB subvariants. In September 2023, the Food and Drug Administration (FDA) of the United States authorized the monovalent XBB.1.5 vaccine booster. The XBB.1.5 vaccine booster induced neutralizing antibodies against the XBB.1.5, XBB.1.16, XBB.2.3, XBB.1.16.6, and EG.5.1 subvariants in mice and non-human primates (Stankov et al., 2024; Patel et al., 2023). Importantly, the XBB.1.5 vaccine booster significantly increased virus-neutralizing antibodies against not only XBB.1.5 but also currently circulating JN.1 in humans (Wang et al., 2024).

Mouse models are pivotal for understanding disease and for the preclinical evaluation of new interventions such as vaccines and monoclonal antibodies (Rosenfeld et al., 2021; van Oosten et al., 2022). The K18-hACE2 mouse is a well-established clinical model for SARS-CoV-2 infection (Dong et al., 2022). The K18-hACE2-transgenic mouse model expresses hACE2 under the human keratin 18 (K18) promoter. This model develops a robust respiratory disease that majorly recapitulates severe COVID-19 symptoms in humans, including lung pathology and excessive inflammation (Natekar et al., 2022; Stone et al., 2021).

However, the comprehensive investigation of XBB.1.5 virulence in K18-hACE-2 mice has not been performed yet. Therefore, the main objective of this study is to evaluate the SARS-CoV-2 XBB.1.5 pathogenesis in K18-hACE-2 transgenic mice. We monitored clinical symptoms and evaluated viral burden and tropism in K18-hACE-2 mice. We evaluated lung pathology upon XBB.1.5 infection using histopathological analysis and the cellular immune response in the lungs via flow cytometric analysis. In addition, we assessed XBB.1.5-induced inflammatory host response in the lungs. Taken together, our results indicate that XBB.1.5 infection results in severe pulmonary disease in K18-hACE2 mice.

## 2 Materials and methods

### 2.1 Cells

Cercopithecus aethiops Kidney Epithelial Cells Expressing Transmembrane Protease, Serine 2 and Human Angiotensin-Converting Enzyme 2 (Vero E6-TMPRSS2-T2A-ACE2 cells, BEI Resources, NIAID, NIH, NR-54970) were cultured at 37°C in Dulbecco’s modified Eagle’s medium (DMEM) supplemented with 10% fetal bovine serum (FBS) and 1% penicillin–streptomycin.

### 2.2 Virus stocks

We obtained SARS-CoV-2, hCoV-19/USA/MD-HP40900/2022 (XBB.1.5) from BEI Resources (NR-59104) and generated all virus stocks in Vero E6-TMPRSS2-T2A-ACE2 cells. Virus stocks were titred by plaque assay and 10^5^ plaque-forming units (PFU) dilution was used for the animal experiments. We performed all virus experiments in approved biosafety level 3 (BSL-3) facilities at Georgia State University.

### 2.3 *In vivo* mouse challenge experiments

We performed the infectious SARS-CoV-2-related *in vivo* mouse experiments in Animal Biosafety Level 3 laboratory (ABSL-3), carrying them out in accordance with the recommendations in the Guide for the Care and Use of Laboratory Animals of the National Institutes of Health. The protocols were approved by the Institutional Animal Care and Use Committee at Georgia State University (A20044). The K18-hACE-2 mice were purchased from the Jackson Laboratory (Bar Harbor, ME, USA). We intranasally inoculated 6-week-old mice with 10^5^ PFU of SARS-CoV-2 (XBB.1.5) or PBS (Mock) under anesthesia and maintained by isoflurane. Roughly equal numbers of male and female mice were used for each experiment. We weighed the animals and monitored their activity, breathing, and neurological signs daily. Independently, we euthanized mice at days 3 and 5 post-inoculation, anesthetizing them with isoflurane followed by perfusion with cold 1X PBS. The lungs, brain, nasal turbinate, kidney, intestines, and olfactory bulb were collected and flash-frozen or fixed in 2-methylbutane or 4% paraformaldehyde (PFA), respectively, for further analysis (Natekar et al., 2022; Stone et al., 2021).

### 2.4 Infectious virus titration using the plaque assay

The tissues harvested from the animals were weighed and homogenized using a Fisherbrand™ Bead Mill 24 Homogenizer (Fisher Scientific, Cat # 15-340-163) and 1.4 mm ceramic beads (Fisher Scientific, Cat # 15-340-153) for 30 s, followed by centrifugation at 10,000 rpm for 10 min. The tissue homogenates were stored at −80°C until use. We measured the virus titers in the tissue homogenates using a plaque assay with Vero E6-TMPRSS2-T2A-ACE2 cells. Briefly, the cells were seeded at a density of 2 × 10^5^ cells per well in six-well tissue culture plates for 3 days to form monolayers. To titrate the infectious virus, we serially diluted the tissue homogenates 10-fold, starting at 1:10, in the cell infection medium and applied them to monolayered Vero E6-TMPRSS2-T2A-ACE2 cells for 1 h at 37°C with gentle rocking every 15 min. After inoculation, cells were overlaid with 1% low-melting agarose. Forty-eight hours after virus inoculation, plates were stained with 2% neutral red in 1% low-melting agarose for visualizing plaque formation. The number of plaques was counted and used to calculate the PFU/gram of tissue (Natekar et al., 2022; Stone et al., 2021; Kumari et al., 2021).

### 2.5 RNA extraction and reverse transcription quantitative PCR (RT-qPCR)

We weighed the frozen tissue samples harvested from the infected animals and lysed them in RLT buffer (Qiagen, Cat# 79216). Total RNA was extracted from the collected tissue samples using a Qiagen RNeasy Mini kit (Qiagen, Cat# 74104) following the manufacturer’s instructions. We measured the viral RNA levels using primers specific for the SARS-CoV-2 N gene (Integrated DNA Technologies, Cat#10006713) and SsoAdvanced Universal Probes Supermix (Bio-Rad, Cat#1725284). We calculated the viral genome copy numbers using a standard curve and expressed the values in genome copies/μg of total RNA. Gene expression of IL6, IRF7, CXCL10, and ZBP1 were determined using SsoAdvanced™ Universal SYBR^®^ Green Supermix (Bio-Rad, Catalog # 1725271). We calculated the fold changes for the genes against the mock-infected samples after normalizing them to GAPDH (Auroni et al., 2023) (see Table 1).

**Table 1:** Primer sequences used for RT-qPCR.

Gene (accession no.)	Forward primer sequence (5′->3′)	Reverse primer sequence (5′->3′)
IL-6 (NM_000600)	CCAGGAGCCCAGCTATGAAC	CCCAGGGAGAAGGCAACTG
IRF7 (NM_016850.3)	CCCCAGGATCATTTCTGGCA	AGGGTTCCTCGTAAACACGG
CXCL10 (NM_021274)	GGTCTGAGTCCTCGCTCAAG	GTCGCACCTCCACATAGCTT
ZBP1 (NM_001139519)	GGCAGAAGCTCCTGTTGACT	CTGTCCTCCTTCTTCAGGCG

### 2.6 Immunofluorescence staining

We harvested the lungs after cardiac perfusion with 1X PBS and fixed them in 4% PFA. Tissue sections were stained with hematoxylin and eosin (H&E) for histopathological evaluation (Abcam, Cat# ab245880). The tissue sections were also incubated with anti-SARS-CoV-2 Nucleocapsid protein monoclonal antibody (GeneTex (HL344), Cat# GTX635679) and DsRNA (J2) mouse antibody (Absolute Antibody, Cat# Ab01299-2.0) (Rothan et al., 2022) overnight at 4°C, followed by incubation with Alexa Fluor 488 Anti-Rabbit (Life Technologies, Cat#A11008) and Alexa Fluor 594 Goat anti-Mouse IgG (Life Technologies, Cat# A11005) antibodies for 30 min at room temperature. In addition, we incubated the lung tissue sections with CD45-Alexa Fluor^®^ 488 (Cell Signaling Technology, Cat# 59572) and Anti-Actin *α*-Smooth Muscle-Cy3™ antibodies (Sigma, Cat# C6198) overnight at 4°C. We mounted the stained sections with Prolong™ Glass Antifade Mountant with NucBlue™ StainDAPI (Thermo Fisher Scientific, Cat# P36981). We acquired the images using a Zeiss LSM980 confocal microscope with ×10 (tiles) and ×20 objectives, then analyzed them using the ZEN 3.8 Blue software (Zhang et al., 2023).

### 2.7 Flow cytometry analysis

For the cytometric analysis of the lungs and spleens, we anesthetized the animals using isoflurane, followed by 1X PBS cardiac perfusion. Spleen and lung single-cell suspensions were generated using the gentle MACS tissue dissociator (Miltenyi Biotec, Cat#130-093-235) following the manufacturer’s instructions. We incubated the spleen and lung single-cell suspensions with Fc Block antibody (BD Pharmingen) in BD FACS™ Pre-Sort Buffer (BD Biosciences) for 10 min at room temperature before staining. We incubated the cells with antibodies against the following markers: FITC Rat Anti-Mouse CD45 (BDB553080), APC Rat Anti-Mouse CD3 (BDB565643), PE Rat Anti-Mouse CD4 (BDB553730), PerCP-Cy5.5 Rat Anti-Mouse CD8β (DB567597), APC-Cy™7 Rat Anti-Mouse CD11b (BD Pharmingen, Cat# 561039), PE-Texas Red CD11c (Thermofisher Scientific, Cat# MCD11C17), and fixable Viability Stain 575 V (BD Biosciences). We stained the cells for 30 min on ice, then washed and fixed them (eBioscience) according to the manufacturer’s instructions. We acquired flow cytometry data on a BD LSRFortessa™ Cell Analyzer and used the FlowJo software for analysis (Roe et al., 2012).

### 2.8 Cytokine and chemokine protein measurements

Lung tissues were homogenized using a Fisherbrand™ Bead Mill 24 Homogenizer (Fisher Scientific, Cat # 15–340-163) and 1.4 mm ceramic beads (Fisher Scientific, Cat # 15–340-153) for 30 s, followed by centrifugation at 10,000 rpm for 10 min. We analyzed the lung homogenates for cytokines and chemokines using the Milliplex Mouse Cytokine/Chemokine Magnetic Bead Panel (Millipore Sigma, Cat# MCYTMAG70PMX25BK) (Basu et al., 2024). The sample concentrations were calculated using the Belysa^®^ Immunoassay Curve Fitting Software (Millipore Sigma).

### 2.9 Statistical analysis

The statistical analyses were performed using the GraphPad Prism software, version 9. Results at *p*-values of *p* < 0.05 were considered statistically significant. We used one-way analysis of variance (ANOVA) followed by Tukey’s multiple comparisons test or the non-parametric Kruskal–Wallis test followed by Dunnett’s multiple comparisons test to compare multiple groups. We used an unpaired Student’s *t*-test to compare the two groups.

## 3 Results

### 3.1 XBB.1.5 infection in K18-hACE2 mice

**Figure 1:**
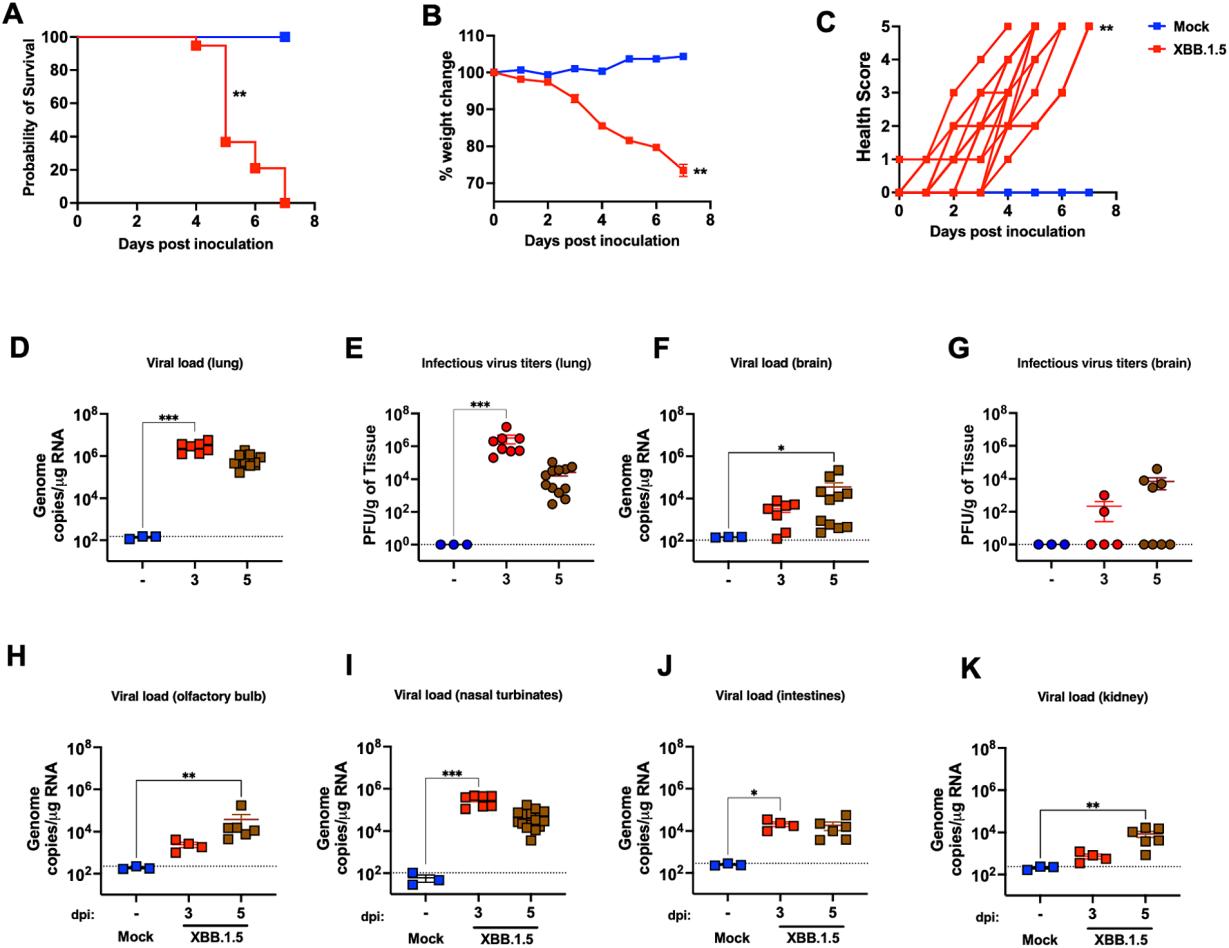
XBB.1.5 infection in K18-hACE-2 mice. Six-week-old male and female K18-hACE-2 mice were inoculated intranasally with 10^5^ PFU SARS-CoV-2 (XBB.1.5) or PBS (Mock). **(A)** Kaplan–Meier survival curve for SARS-CoV-2-infected K18-hACE-2 mice (three independent experiments; XBB.1.5- and mock-infected mice; *n* = 19 and 6, respectively; Log-rank (Mantel-Cox) test; *p* = 0.0012). **(B)** Weight change was monitored for 7 days post-infection (dpi). Mice were weighed daily (three independent experiments; XBB.1.5- and mock-infected mice *n* = 19 and 6, respectively; two-tailed unpaired *t*-test; *p* = 0.0026). Weights are expressed as the percentage of initial body weight (day 0). The bars indicate the mean ± SEM. **(C)** Clinical score (0 = normal; 1 = ruffled fur and slight hunch; 2 = ruffled fur, hunched, and slow; 3 = ruffled fur, hunched, slow, and rounded; 4 = ruffled fur, hunched, slow, rounded, shaking, and labored breathing; 5 = found dead or reached endpoint; two-tailed unpaired *t*-test; *p* = 0.0036). **(D)** Viral load in the lungs was quantified by RT-qPCR. The data are expressed on the log scale of the genomic copies/μg of RNA. **(E)** Infectious virus titers in the lungs were quantified by plaque assay. The data are expressed as the PFU/g of tissue. **(F)** Viral load in the brain was quantified by RT-qPCR. The data are expressed on the log scale of the genomic copies/μg of RNA. **(G)** Infectious virus titers in the brain were quantified by plaque assay. The data are expressed as the PFU/g of tissue. **(H–K)** Viral RNA copy number in the olfactory bulb, nasal turbinate, small intestines, and kidney were determined at 3 and 5 dpi using RT-qPCR. The data are expressed on the log scale of the genomic copies/μg of RNA. **(D–K)** Statistical significance determined by a one-way ANOVA followed by Tukey’s multiple comparisons or the Kruskal–Wallis test, followed by Dunn’s multiple comparisons test (*p < 0.05; ** *p* < 0.01; *** *p* < 0.001, **** *p* < 0.0001). Each point represents an individual mouse. The bars indicate the mean ± SEM.

To characterize K18-hACE2 mouse susceptibility to the Omicron subvariant XBB.1.5, we challenged 6-week-old K18-hACE2 mice intranasally with 10^5^ PFU of XBB.1.5 or PBS (Mock) and monitored the animals daily for any sign of illness. All PBS-inoculated mice remained healthy. As early as day 3 post-inoculation, K18-hACE-2 mice exhibited significant weight loss and clinical signs (e.g., ruffled fur, lethargy, and hunched posture). From day 4 post-infection (dpi), mice displayed more severe symptoms (e.g., labored breathing and slow movement). Infection resulted in a 100% mortality rate in K18-hACE-2 mice by day 7 post-inoculation (Figures 1A–C).

Furthermore, in independent experiments, K18-hACE-2 mice were inoculated intranasally with 10^5^ of the XBB.1.5 or PBS (Mock) and euthanized at 3 and 5 dpi. Viral RNA levels in the lungs were evaluated using RT-qPCR. We detected high SARS-CoV-2 RNA levels in the infected lungs at 3 and 5 dpi (Figure 1D). In addition, we quantified the infectious virus titers in the lung homogenates collected at 3 and 5 dpi using a plaque assay, observing consistent results with those of the RT-qPCR. We detected high levels of infectious virus in the infected lungs at 3 dpi with a slight reduction by 5 dpi (Figure 1E).

### 3.2 XBB.1.5 tropism and multiorgan infection

SARS-CoV-2 neuroinvasion is observed in K18-hACE-2 mice (Kumari et al., 2021; Winkler et al., 2020; Stewart et al., 2023; Oh et al., 2024). In addition, K18-hACE2 mouse-derived primary neuronal cultures were permissible for SARS-CoV-2 infection (Rothan et al., 2022). A recent report suggested the increased neurotropic potential of BA.5 and XBB isolates (Stewart et al., 2023). Therefore, we evaluated XBB.1.5 neurotropic potential in K18-hACE-2 mice. We detected viral RNA and infectious virus in the brain of a subset of infected K18-hACE2 mice (Figures 1F,G). We detected viral RNA in the olfactory bulb of all the animals, indicating productive infection within the olfactory system (Figure 1H). Additionally, high viral RNA levels were detected in the nasal turbinate (Figure 1I).

To further elucidate SARS-CoV-2 tissue tropism, SARS-CoV-2 RNA levels were evaluated in the kidneys and small intestines. Accumulating evidence suggests the intestines as a target organ for SARS-CoV-2, and gastrointestinal symptoms have been extensively reported in patients with COVID-19. In addition, SARS-CoV-2 was isolated from stool samples collected from patients with severe COVID-19 (Xiao et al., 2020). Similarly, we detected viral RNA in the small intestines of infected-K18-hACE-2 mice (Figure 1J). Acute kidney injury is observed in a subset of critically ill patients with COVID-19 along with the presence of SARS-CoV-2 in their urine samples (Gabarre et al., 2020; Sun et al., 2020). Therefore, we evaluated SAR-CoV-2 presence in the kidneys collected from K18-hACE-2 mice upon XBB.1.5 challenge. Consistent with the aforementioned studies, we detected viral RNA in the kidneys of K18-hACE-2 mice with a significant increase by 5 dpi (Figure 1K). In summary, our data demonstrate the direct multiorgan XBB.1.5 invasion in K18-hACE-2 mice.

### 3.3 SARS-CoV-2 antigen expression and induced lung pathology

**Figure 2:**
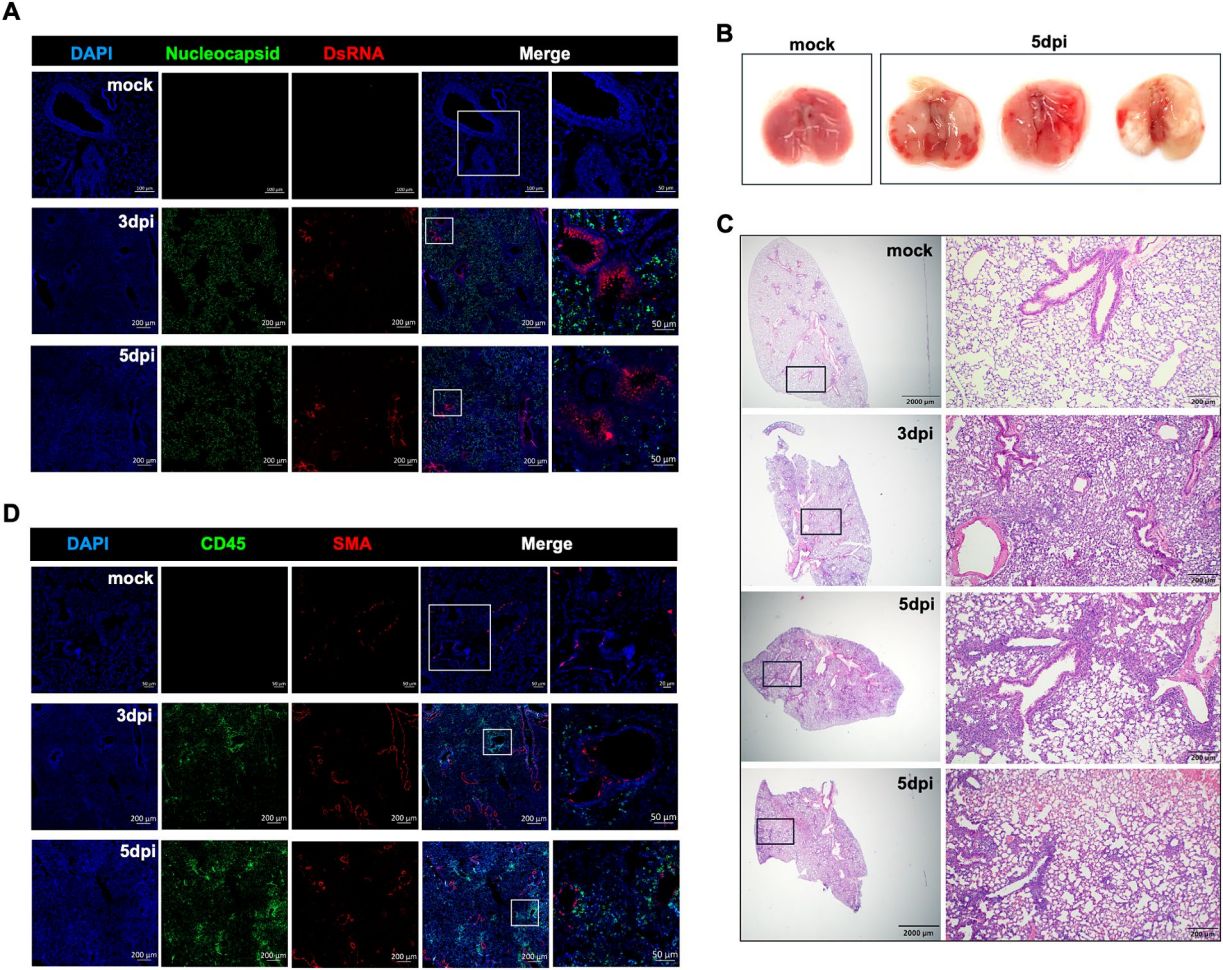
Histopathological analysis of XBB.1.5 infection in K18-hACE2 mice. **(A)** Representative images of dual immunofluorescence-stained lung tissues. Lung samples were collected from mock-infected control and at days 3 and 5 post XBB.1.5 infection and labeled with the SARS-CoV-2 nucleocapsid protein (green), DsRNA (red), and nuclei (blue). Scale bars for mock-infected control lung: 100 μm and 50 μm on the original and high-magnification images, respectively. Scale bars for XBB.1.5-infected lungs: 200 μm and 50 μm on the original and high-magnification images, respectively (A representative image is shown for each group). **(B)** Images of whole lungs collected from mock-infected control (*n* = 1) and at 5 dpi with XBB.1.5 (*n* = 3). **(C)** Representative images of hematoxylin and eosin (H&E)-stained lung sections collected from mock-infected control and at 3 or 5 dpi with XBB.1.5 (A representative image is shown for mock and 3 dpi; 2 representative images from 2 individual mice are shown for 5 dpi; Scale bars: 2,000 μm and 200 μm on the original and high-magnification images, respectively). **(D)** Representative images of dual immunofluorescence-stained lung tissues collected from mock-infected control and at days 3 and 5 post XBB.1.5 infection. Lung samples were labeled with CD45-Alexa Fluor^®^ 488 (green), Anti-Actin *α*-Smooth Muscle-Cy3™ (red), and DAPI (blue). Scale bars for mock-infected control lung: 50 μm and 20 μm on the original and high-magnification images, respectively. Scale bars for XBB.1.5-infected lungs: 200 μm and 50 μm on the original and high-magnification images, respectively (A representative image is shown for each group).

Next, infected lung tissues were analyzed for antigen distribution and double-stranded RNA presence. Consistent with the viral titer data, the infected lung tissue samples contained abundant SARS-CoV-2 nucleocapsid protein at 3 and 5 dpi. As an RNA virus, SARS-CoV-2 produce dsRNA early during the infection cycle. Therefore, we further assessed viral replication in the infected lungs using anti-dsRNA immunofluorescence labeling. We detected a robust dsRNA signal in the conducting airway epithelia and the bronchioles with an intense signal at 3 dpi that waned slightly by 5 dpi (Figure 2A).

Gross pathology images revealed multifocal and fibrotic lesions in the lungs collected at 5 dpi (Figure 2B). Next, we evaluated the virus-induced lung injury through the histological analysis of the lung tissue at 3 and 5 dpi, revealing markedly altered alveolar ducts and sacs as early as 3 dpi. Notably, at 5 dpi, the infected lungs displayed severe pathology characterized by multifocal lesions with abundant immune cell infiltration into the alveolar spaces, perivascular cuffing, alveolar space consolidation, vascular congestion, and hypercellular thickening of the alveolar septae (Figure 2C).

**Figure 3:**
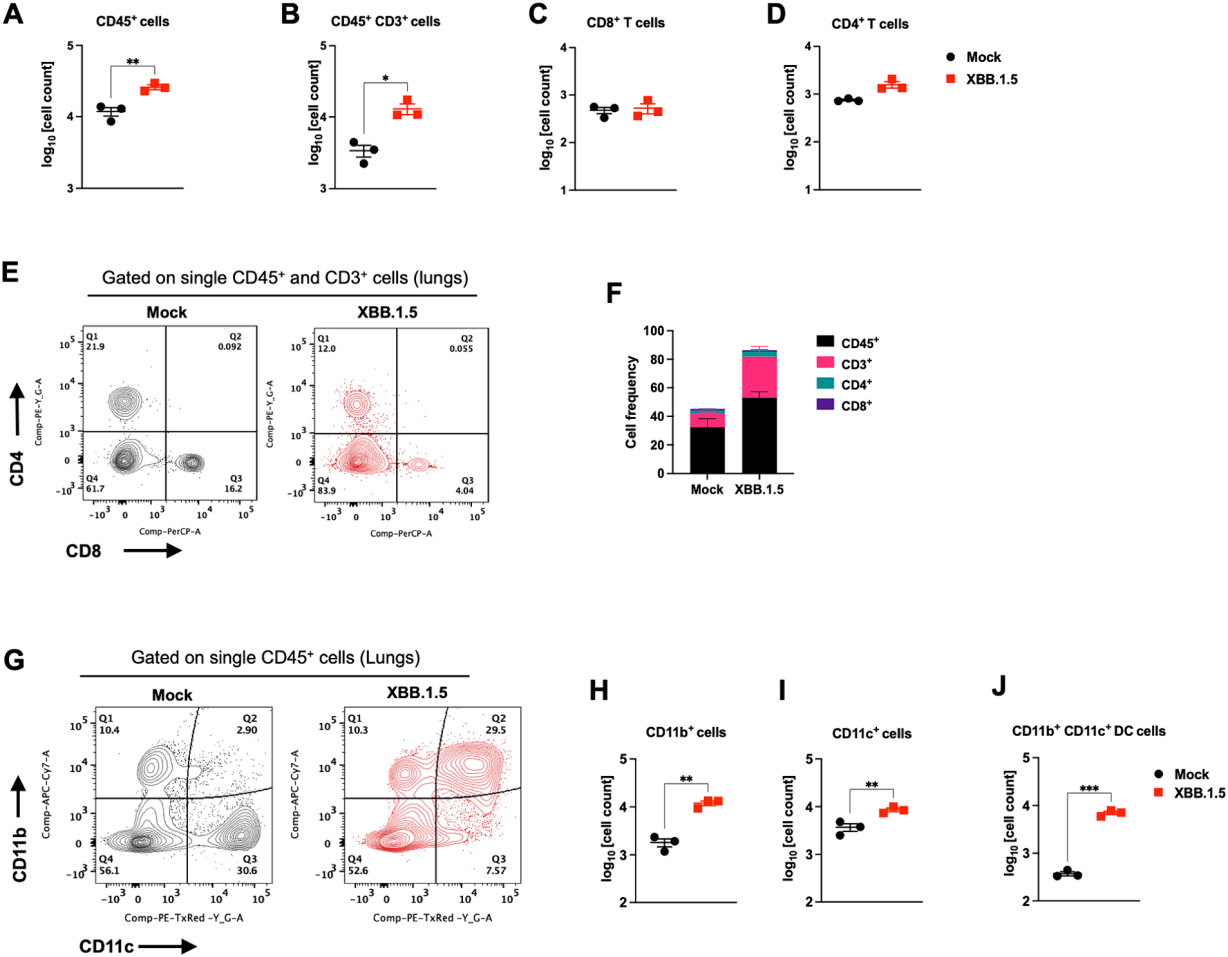
Cellular immune response to XBB.1.5 infection. Flow cytometric analysis of lung tissues in K18-hACE2 mice following intranasal inoculation with XBB.1.5 or mock infection. **(A)** CD45^+^ cell numbers in mock- and XBB.1.5-infected lungs. **(B)** CD45^+^ CD3^+^ cell numbers. **(C)** CD8^+^ T-cell numbers. **(D)** CD4^+^ T-cell numbers. **(E)** Representative expression of CD4 and CD8 visualized by surface staining on CD45^+^ CD3^+^ cells in mock- and XBB.1.5-infected lungs. **(F)** Frequency of CD45^+^, CD3^+^, CD8^+^ T, and CD4^+^ T cells in the lungs. **(G)** Representative expression of CD11c^+^ and CD11b^+^ visualized by surface staining on CD45^+^ cells in mock- and XBB.1.5-infected lungs. **(H)** CD45^+^ CD11b^+^ cell numbers. **(I)** CD45^+^ CD11c^+^ cell numbers. **(J)** CD45^+^ CD11c^+^ CD11b^+^ cell numbers. Each point represents an individual mouse. The bars indicate the mean ± SEM. *n* = 3 both for mock- and 5 dpi XBB.1.5-infected animals; two-tailed unpaired *t*-test (*p < 0.05; ** *p* < 0.01; *** *p* < 0.001, **** *p* < 0.0001).

In response to infection, the lung resident immune cell activation orchestrates CD45^+^ leukocyte recruitment from the circulation. Therefore, we deployed CD45-specific antibody labeling of the lungs harvested at 3 and 5 dpi. Our immunofluorescence analysis revealed abundant CD45-positive immune cell infiltrates around the blood vessels and within the alveolar spaces (Figure 2D). To further evaluate the components of the cellular immune response in the lungs upon XBB.1.5 infection, we performed flow cytometric analysis on lung and spleen homogenates on day 5 after intranasal virus inoculation (Supplementary Figures S1, S2). Consistent with the histopathological analysis, we detected a significant increase in the number of CD45^+^ immune cells in the infected lungs (Figure 3A). Despite the significant increase in CD45^+^ CD3^+^ cells in the lungs (Figure 3B), no significant increase was observed in the CD3^+^CD4^+^ and CD3^+^CD8^+^ T-cell populations in the infected lungs (Figures 3C–F). By 5 dpi, we observed a significant increase in CD45^+^ CD11b^+^ and CD45^+^ CD11c^+^ immune cells in the lungs. The cellular infiltrates in the infected lungs were mainly composed of CD11b^+^CD11c^+^ dendritic cells (Figures 3G–J), consistent with previous studies describing a significant increase in CD11b^+^CD11c^+^ dendritic cells in SARS-CoV-2-infected lungs and the BAL fluid (Winkler et al., 2020). Finally, we observed no significant difference in the splenic CD3^+^CD4^+^ and CD3^+^CD8^+^ T-cell populations (Supplementary Figures S2A,B).

### 3.4 XBB.1.5 infection causes excessive lung inflammation

**Figure 4:**
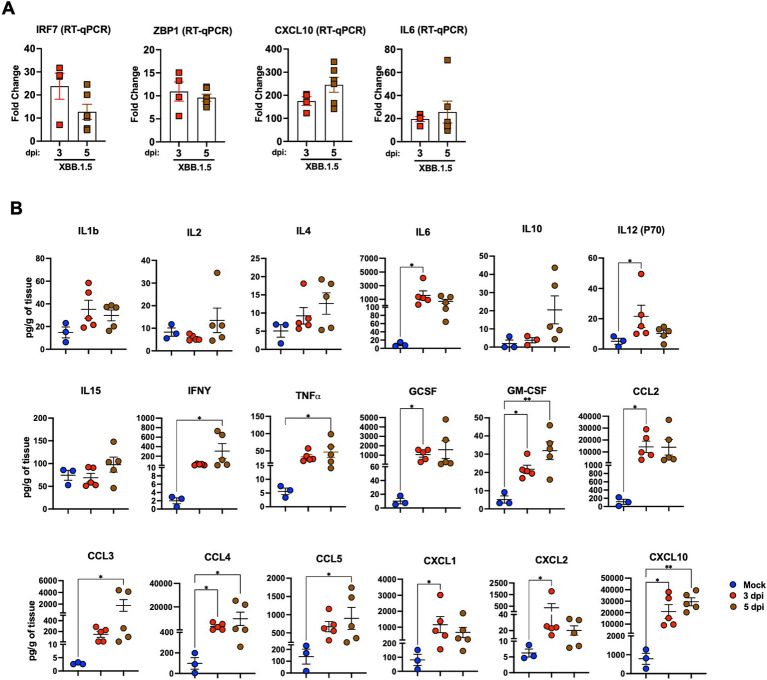
Cytokine and chemokine levels in the lungs upon XBB.1.5 infection. **(A)** RNA levels of the indicated genes determined in lung homogenates using RT-qPCR. The fold change in gene expression was determined for IRF7, ZBP1, IL6, and CXCL10. Two independent experiments; *n* = 6 animals per group. The bars indicate the mean ± SEM. The fold change was calculated in the XBB.1.5-infected lungs after comparison with mock-infected controls and normalization to GAPDH levels in each sample. **(B)** Cytokine and chemokine protein levels in the lungs of mock- and XBB.1.5-infected animals at 3 and 5 dpi. The middle bar indicates the mean ± SEM; *n* = 3 animals for mock-infected, *n* = 5 for XBB.1.5-infected animals at 3 and 5 dpi. Statistical significance was determined by a one-way ANOVA followed by Tukey’s multiple comparisons or the Kruskal–Wallis test, and Dunn’s multiple comparisons test (*p < 0.05; ** *p* < 0.01; *** *p* < 0.001, **** *p* < 0.0001). Each point represents an individual mouse. The bars indicate the mean ± SEM.

First, we analyzed the expression level of Interferon Regulatory Factor 7 (IRF7), a key player in the interferon signaling pathway. We detected significant upregulation of IRF7 in the infected lungs with a 23.83- and 12.72-fold increase at 3 and 5 dpi, respectively. Next, we evaluated the gene expression of Z-DNA binding protein (ZBP1), implicated in cell death pathways upon viral infections (Hao et al., 2022). At 3 dpi, ZBP1 average expression levels increased to approximately 10.94-fold and to 9.61-fold by 5 dpi. Consistently, SARS-CoV-2 Z-RNA induces the ZBP1-RIPK3 pathway, yielding an enhanced inflammatory response (Li et al., 2023; Karki et al., 2022). Furthermore, proinflammatory cytokine and chemokine secretions are reportedly dysregulated in patients with severe COVID-19 (Zhou Z. et al., 2020). Cytokine profiling of the sera of patients with COVID-19 and the transcriptional analysis of their BAL fluid revealed increased IL-6, G-CSF, CXCL10, CCL2, CCL3, and TNF-*α* levels, correlating with disease severity and progression (Chen et al., 2020; Yang et al., 2020). Therefore, we evaluated IL-6 and CXCL10 expression levels in lung homogenates. XBB.1.5 infection resulted in an average 19.64- and 25.72-fold increase in IL-6 levels at 3 and 5 dpi, respectively. The transcription level of the CXCL10 gene was significantly upregulated with a 174.73- and 245.69-fold increase at 3 and 5 dpi, respectively (Figure 4A).

Next, using a multiplex immunoassay, we quantified the protein levels of proinflammatory cytokines and chemokines in mock- and XBB.1.5-infected lung homogenates. Compared with mock samples, we detected significant increased levels of several proinflammatory cytokines, e.g., interleukin-1b (IL-1b), interleukin-6 (IL-6), interleukin-12 (IL12-p70), and interleukin-15 (IL-15). Interferon-y (IFN-y), tumor necrosis factor α (TNFα), granulocyte colony-stimulating factor (G-CSF), and granulocyte-macrophage colony-stimulating factor (GM-CSF) levels significantly increased in infected lungs. Moreover, we detected a significant increase in C–C motif (CCL2, CCL3, CCL4, and CCL5) and C–X–C motif (CXCL1, CXCL2, and CXCL10) chemokines (Figure 4B). Taken together, our data revealed excessive lung inflammation, probably due to increased viral replication in the lungs of XBB.1.5-infected K18-hACE2 mice.

## 4 Discussion

In this study, we evaluated Omicron subvariant XBB.1.5-induced pathogenicity in K18-hACE-2-transgenic mice. Early Omicron subvariants are reportedly less pathogenic in mice, hamsters, and humans (Nyberg et al., 2022; Ward et al., 2022; Halfmann et al., 2022; Hui et al., 2022). We have previously shown that infection with Omicron, B.1.1.529, resulted in attenuated disease with limited viral replication in the lungs compared to pre-Omicron VOCs including B.1.1.7, B.1.351, and B.1.617.2. Additionally, we have previously shown that infection with B.1.1.529 resulted in only 50% mortality in K18-hACE-2 mice (Natekar et al., 2022). Herein, we report that unlike B.1.1.529, XBB.1.5 infection resulted in significant weight loss and 100% mortality rate and caused severe pulmonary disease in K18-hACE-2 mice. XBB.1.5 infection also resulted in significantly high levels of viral RNA and infectious virus in the infected lungs. The levels of infectious virus in the lungs of XBB.1.5-infected mice were approximately 100-fold higher than in the lungs of B.1.1.529-infected mice, despite using the same inoculation titers for virus challenge (Natekar et al., 2022). Interestingly, infectious virus was detected in the brain of a subset of XBB.1.5-infected mice. These observations suggest that lethal XBB.1.5 infection is more consistent with lung infection rather than brain infection. In addition, we detected viral RNA in the olfactory bulb, suggesting viral invasion of the cells within the olfactory system. We also detected significant levels of viral RNA in the peripheral organs. Consistent with previous reports, we detected viral RNA in the small intestines (Xiao et al., 2020). A subset of critically ill patients with COVID-19 reportedly displayed acute kidney injury (Gabarre et al., 2020; Sun et al., 2020). In line with these studies, we detected viral RNA in the kidneys of mice infected with XBB.1.5.

SARS-CoV-2 infection is asymptomatic or mild in most patients with COVID-19 (Wu and McGoogan, 2020). However, age and comorbidity might represent risk factors for severe COVID-19 complications that require hospitalization (Wang D. et al., 2020). In the model of this study, we observed severe COVID-19 symptoms including lung pathology characterized by increased immune infiltrates, perivascular cuffing, and alveolar consolidation upon XBB.1.5 infection. We detected aberrant recruitment and substantial accumulation of CD45-positive cells in the lungs, consistent with observations of patient sample biopsies (Won et al., 2022). In severe COVID-19 cases in humans, T-cell lymphopenia could be observed, characterized by profound CD4 and CD8 lymphocyte reduction (Wang F. et al., 2020; Davitt et al., 2022). In contrast, strong CD4 and CD8 T-cell responses are reportedly associated with low disease severity in individuals with COVID-19 (Grifoni et al., 2020). Consistent with these results, we did not detect any significant increase in the number of CD4^+^ and CD8^+^ T-cell populations either in the lungs or the spleen of the infected K18-hACE-2 mice. Further, we detected a significant increase in the CD11b^+^ CD11c^+^ dendritic cells in the lungs, consistent with previous reports on the cellular response of SARS-CoV-2 infection in K18-hACE2 mice (Winkler et al., 2020). In addition, we detected significantly increased levels of several chemokines and proinflammatory cytokines. The exaggerated immune response in K18-hACE-2 mice, characterized by excessive induction of proinflammatory mediators, might explain in part the severe tissue damage observed in the infected K18-hACE-2 lungs. Although the exact effector mechanism of the enhanced XBB.1.5 virulence in K18-hACE2 mice remains unclear, studies have suggested increased fusion activity and enhanced ACE-2 binding affinity because of the unique mutations present in XBB.1.5 (Yue et al., 2023).

Although the K18-hACE2 mice recapitulate the human symptoms of severe respiratory COVID-19, this model exhibits several limitations. The expression of the hACE2 transgene, driven by the K18 promoter, is independent of the mouse ACE2 expression. Therefore, the expression of hACE2 in K18-hACE2 mice does not recapitulate the human ACE2 expression levels or the exact human ACE2 distribution. In this model, human ACE2 is expressed in different tissues, allowing for the SARS-CoV-2 infection of multiple organs, potentially enhancing disease severity (McCray et al., 2007). In this context, it would be of interest to evaluate the effects of XBB.1.5 infection in more humanized ACE2 models (Ghanam et al., 2022).

In summary, our studies demonstrated the high virulence of the XBB.1.5 subvariant in K18-hACE2-transgenic mice. XBB.1.5 infection resulted in robust viral replication in the lungs, dichotomous brain infection, and the viral invasion of peripheral organs. Our data suggest that combined infection, increased cellular infiltrates, and inflammation in the lungs contributed to the severe pulmonary disease observed in K18-hACE2 mice.

## Data Availability

The original contributions presented in the study are included in the article/Supplementary material, further inquiries can be directed to the corresponding author.

## Supplementary material

SUPPLEMENTARY FIGURE S1 Cellular immune response to XBB.1.5 infection in the lungs. **(A)** Gating strategy and FACS plots of CD4^+^ and CD8^+^ T cells in the lung tissue of K18-hACE2 mice upon intranasal infection with XBB.1.5 or mock. FACS plots are shown for each individual mouse (*n* = 3 both for mock- and 5 dpi XBB.1.5-infected animals). **(B)** Gating strategy and FACS plots of CD11b^+^, CD11c^+^, and CD11c^+^ CD11b^+^ in lung tissues of mock- and XBB.1.5-infected mice. FACS plots are shown for each individual mouse (*n* = 3 per group both for mock- and 5 dpi XBB.1.5-infected animals).

SUPPLEMENTARY FIGURE S2 T-cell response to XBB.1.5 infection in the spleen. **(A)** Gating strategy and FACS plots of CD4^+^ and CD8^+^ T cells in the spleen tissue of K18-hACE2 mice upon intranasal infection with XBB.1.5 or mock. FACS plots are shown for each individual mouse (*n* = 3 per group). **(B)** CD4^+^ and CD8^+^ T-cell numbers in mock- and XBB.1.5-infected spleens, gated on live CD45^+^ CD3^+^ cells (*n* = 3 per group).
