# Supplementary figures and images for: Omicron XBB.1.5 subvariant causes severe pulmonary disease in K18-hACE-2 mice
Source: Front Microbiol. 2024 Oct 2;15:1466980. doi: 10.3389/fmicb.2024.1466980 (PMC11480052; doi:10.3389/fmicb.2024.1466980)

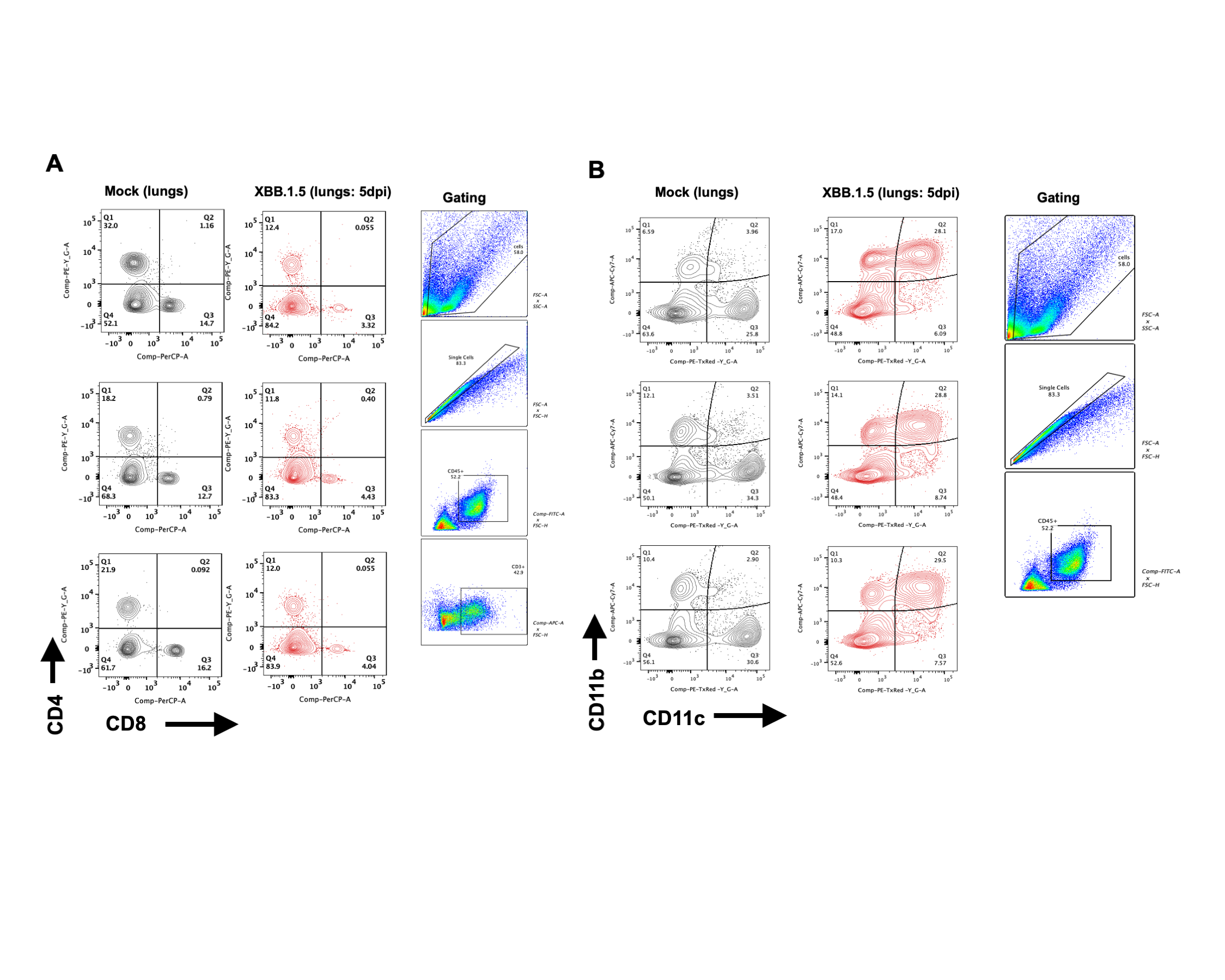

Supplement: SUPPLEMENTARY FIGURE S1 — Cellular immune response to XBB.1.5 infection in the lungs. (A) Gating strategy and FACS plots of CD4+ and CD8+ T cells in the lung tissue of K18-hACE2 mice upon intranasal infection with XBB.1.5 or mock. FACS plots are shown for each individual mouse (n = 3 both for mock- and 5 dpi XBB.1.5-infected animals). (B) Gating strategy and FACS plots of CD11b+, CD11c+, and CD11c+ CD11b+ in lung tissues of mock- and XBB.1.5-infected mice. FACS plots are shown for each individual mouse (n = 3 per group both for mock- and 5 dpi XBB.1.5-infected animals). [file Image_1.TIFF]

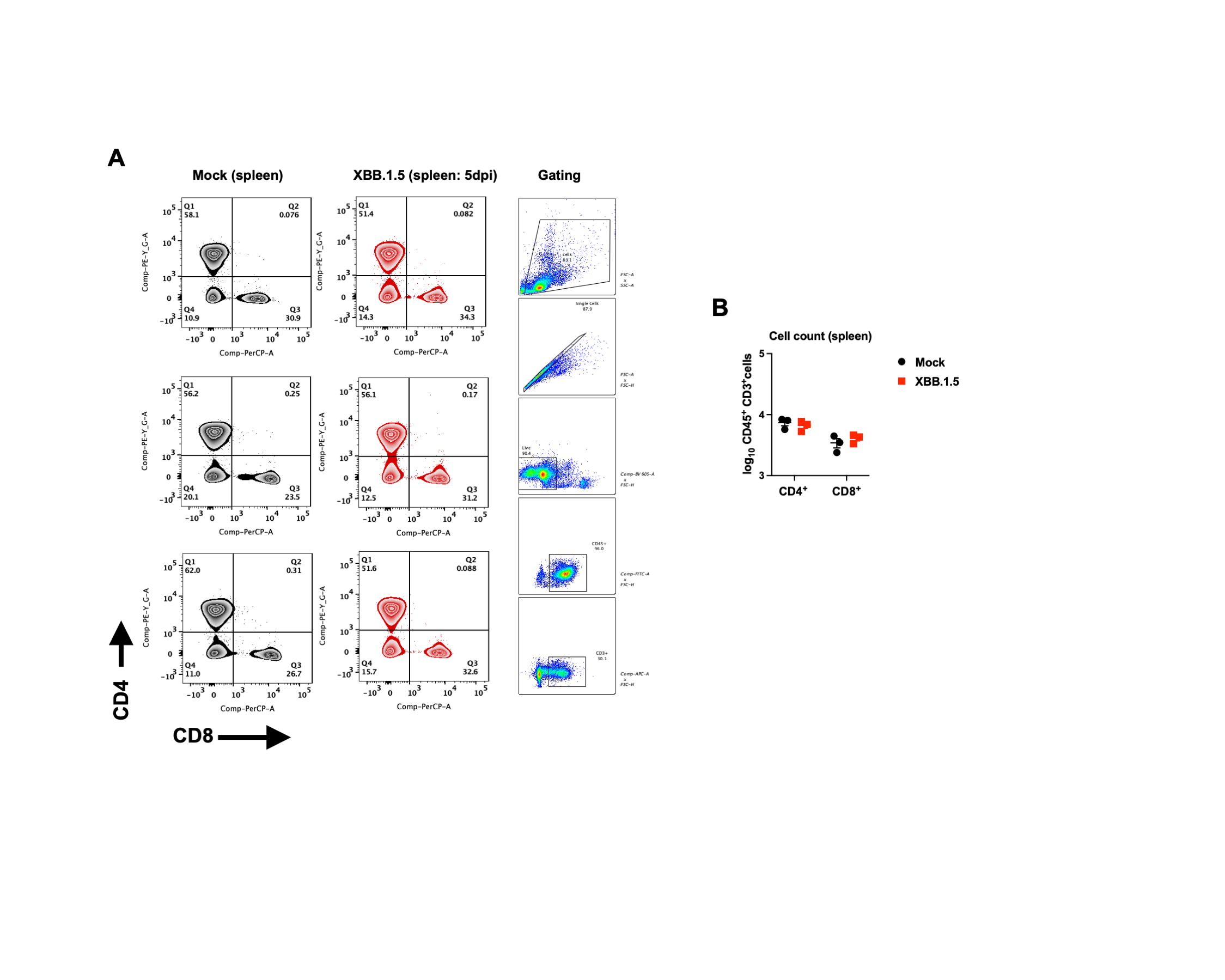

Supplement: SUPPLEMENTARY FIGURE S2 — T-cell response to XBB.1.5 infection in the spleen. (A) Gating strategy and FACS plots of CD4+ and CD8+ T cells in the spleen tissue of K18-hACE2 mice upon intranasal infection with XBB.1.5 or mock. FACS plots are shown for each individual mouse (n = 3 per group). (B) CD4+ and CD8+ T-cell numbers in mock- and XBB.1.5-infected spleens, gated on live CD45+ CD3+ cells (n = 3 per group). [file Image_2.TIFF]
